# Supplementary material for: Optical and physical mapping with local finishing enables megabase-scale resolution of agronomically important regions in the wheat genome
Source: Genome Biol. 2018 Aug 17;19:112. doi: 10.1186/s13059-018-1475-4 (PMC6097218; doi:10.1186/s13059-018-1475-4)
Supplement: Supplementary file 1 — BAC preparation and analysis for physical maps. (DOCX 16 kb) [file 13059_2018_1475_MOESM1_ESM.docx]

## **Additional file 1: BAC preparation and analysis for physical maps**

The chromosome arms for 7AS and 7AL

The short and long arms of chromosome 7A were purified by flow cytometric sorting from ditelosomic lines 7AS and 7AL of wheat cv. Chinese Spring, in which the arms are stably maintained as telocentric chromosomes. For preparation of samples for flow cytometry and chromosome sorting see (17). In total, 6.4x10^6^ 7AS arms, equivalent of 5.2 μg DNA, and 4.8x10^6^ 7AL arms, equivalent of 3.9 μg DNA were obtained. The identity and purity in the sorted fractions was checked by fluorescence *in situ* hybridization using probes for telomeric repeat and GAA repeat (see 17). The 7AS arm was sorted with the purity of 83.4%, while the 7AL arm was sorted with 80.5% purity. High molecular weight DNA prepared from the sorted arms was used to construct arm-specific BAC libraries following established protocols and construct Bionano maps (see 17). To estimate average insert size, 160 BAC clones were randomly selected from each of the libraries and analyzed as described elsewhere (see 17).

## Physical map construction (LTC)

The LTC software (20) was used to establish ordered assemblies of the BAC clones from the DNA fingerprint information in order to select a minimum tiling path (MTP) for sequencing. The following procedure was carried out for 7AS and 7AL independently: a network of “overlaps” was constructed using Sulston score cutoff Xe-10. Five rounds of increased stringency were applied, as well as Q-clones being identified at each step (20). Contigs with less than 5 clones were not included in the final outputs. The statistics for this first version of the physical assembly are summarized below.

| **Chromosome arm** | **# contigs** | **# clones** | **# clones in MTP** | **N50** | **Total length** |
| --- | --- | --- | --- | --- | --- |
| 7AS | 380 | 42,244 | 5,280 | 1.38Mb | 353Mb |
| 7AL | 352 | 47,776 | 5,832 | 1.70Mb | 402Mb |

Following the acquisition of sequence data from the MTP BAC clones and anchoring of the BACs to the high resolution genetic map, the construction of the physical map was repeated using improved LTC software (Z Frenkel unpublished) which utilized the additional information to validate linkages and extend some of the assemblies.
