# Supplementary material for: Optical and physical mapping with local finishing enables megabase-scale resolution of agronomically important regions in the wheat genome
Source: Genome Biol. 2018 Aug 17;19:112. doi: 10.1186/s13059-018-1475-4 (PMC6097218; doi:10.1186/s13059-018-1475-4)
Supplement: Supplementary file 2 — Figures S1–S7. (with legends) for Additional files. (DOCX 5813 kb) [file 13059_2018_1475_MOESM2_ESM.docx]

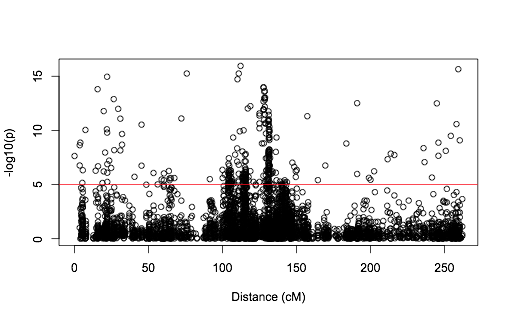


Figure S1. Removal of markers showing excessive segregation distortion (filtering markers with p<1e-5 testing for Mendelian segregation). As indicated in Fig. S1, more than 50% of the 400 markers removed were located in the centromere region


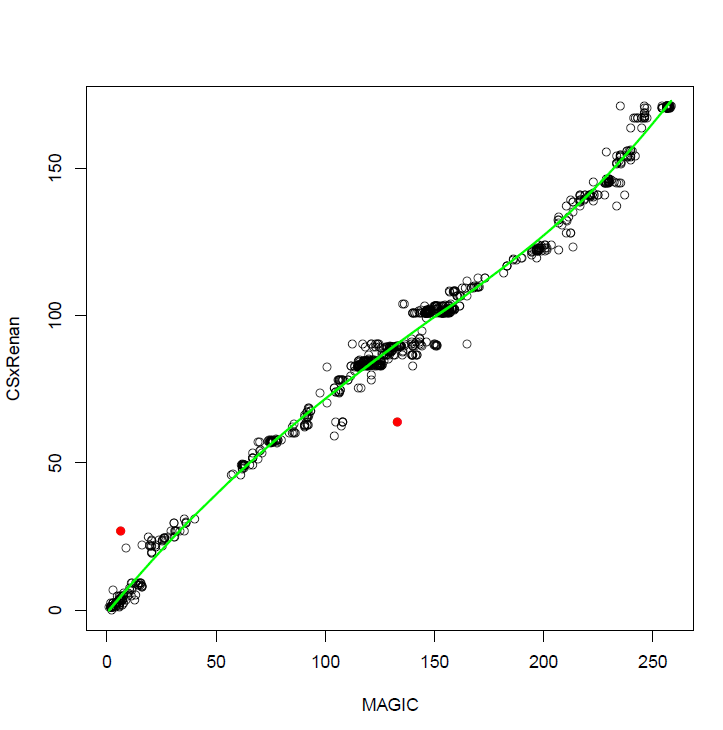


Figure S2

Integrating CS x Renan and MAGIC 7A’s – the two maps were plotted against each to illustrate how consistent the two genetics maps were with respect to each other.


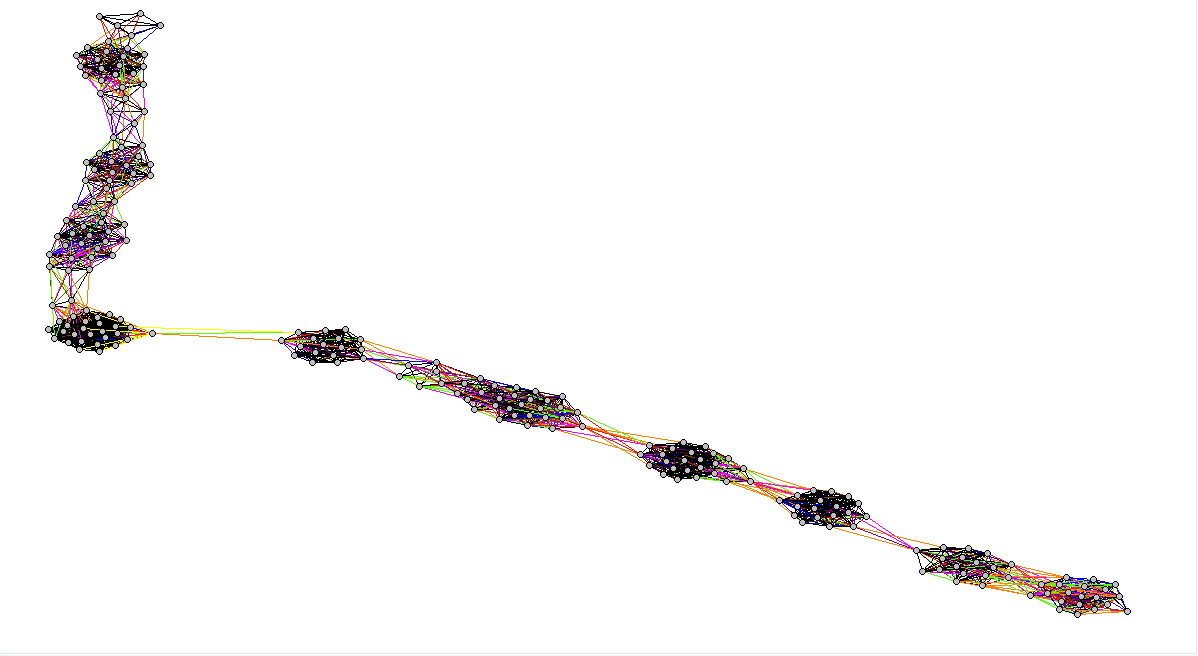


Figure S3: LTC based assembly of the BACs using high resolution fingerprint data. In the alignment of the physical contigs to the MAGIC-7A molecular genetic map, some cases arose where the map suggested no linkage between markers located in a physical contig. If re-examination of the physical contig (example shown) indicated a ‘weak link’ in the physical contig assembly then the assembly was split into ‘a’ and ‘b’ contigs. If the physical contig evidence was unambiguous, the markers were set aside for reconsideration in light of more evidence being obtained.


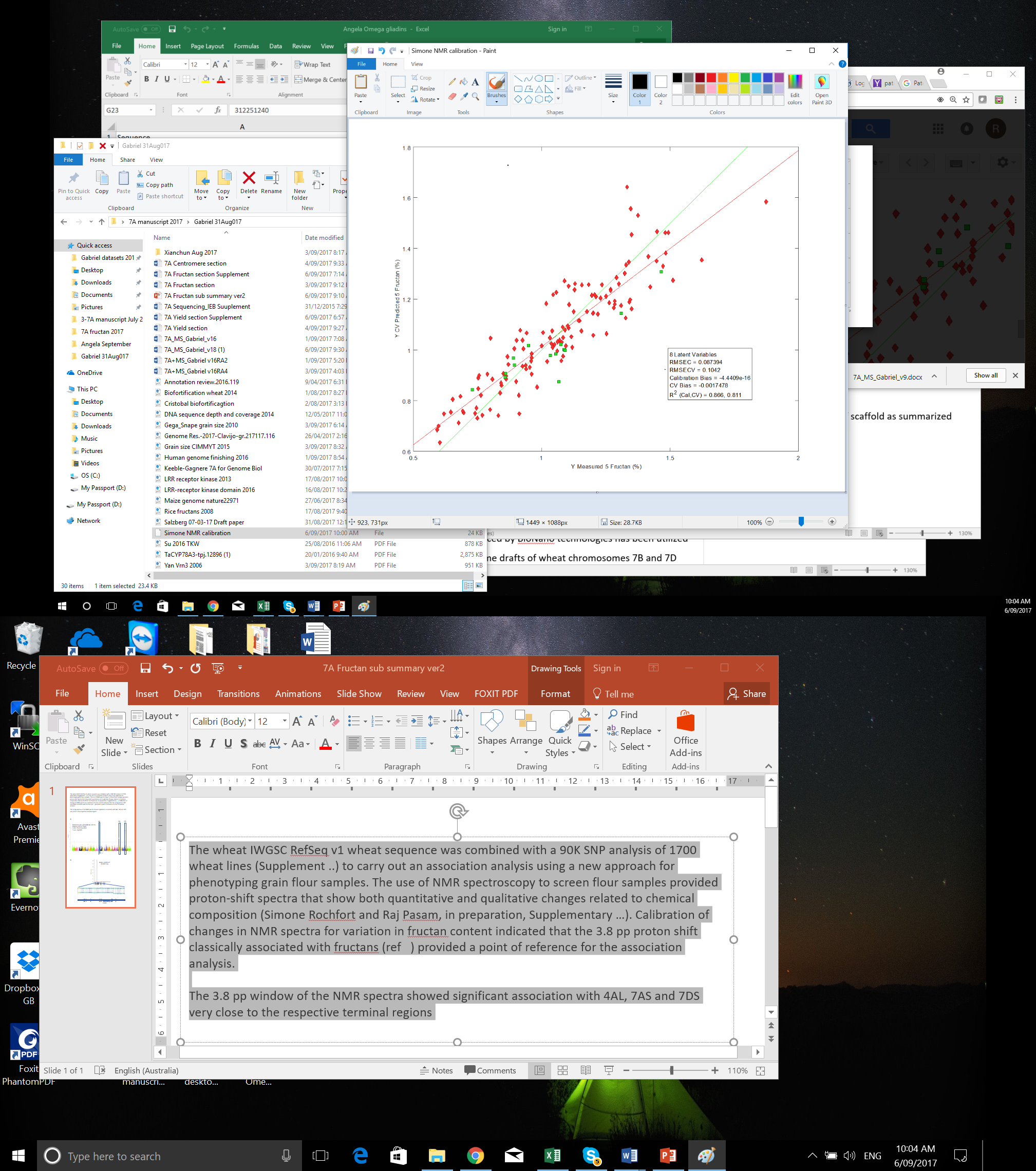


**B**


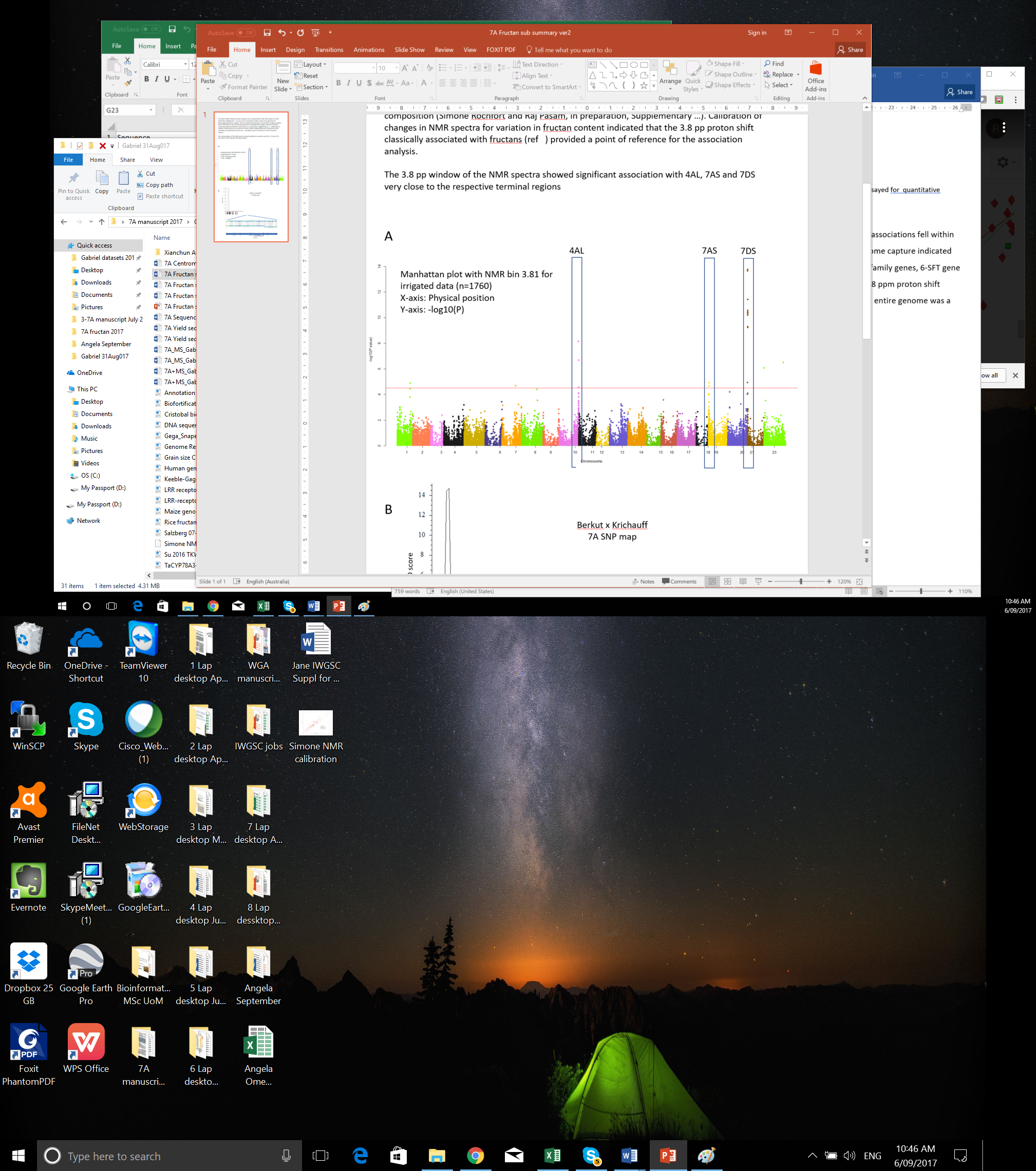


Figure S4

A A total of 155 flour samples from grain produced by a selection of geographically diverse wheat lines were assayed for quantitative change in fructan content using a standard wet chemistry protocol (ref) and variation at the 3.8 ppm proton shift section of the NMR spectra.

B Shows the 3.8 ppm window of the NMR spectra with significant associations of SNPs in 4AL, 7AS and 7DS using standard GWAS analyses where SNPs from exome capture studies of 900 diverse wheat lines were deployed. Using BaysR gave consistent results


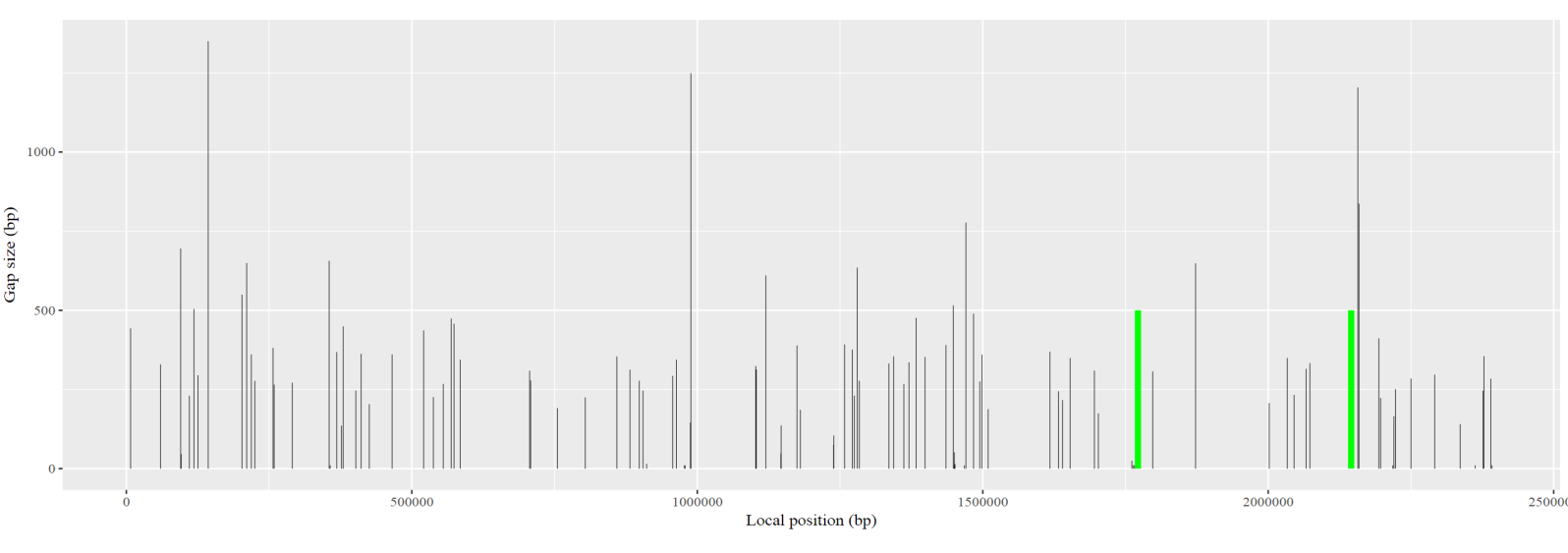


Figure S5. Location of gaps of Ns closed in IWGSC RefSeq are shown with thin black lines. Gaps between IWGSC RefSeq scaffolds, closed in GYDLE sequence, shown with thick green lines.

| **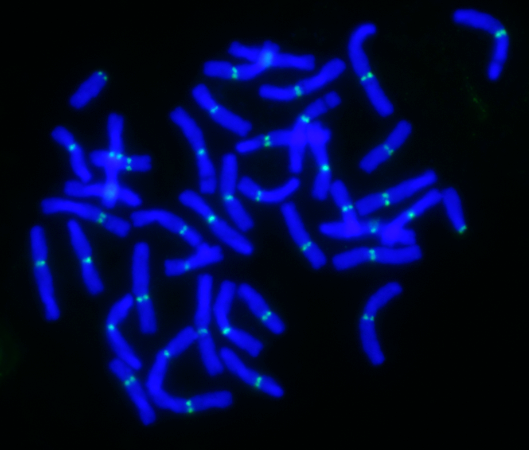** | **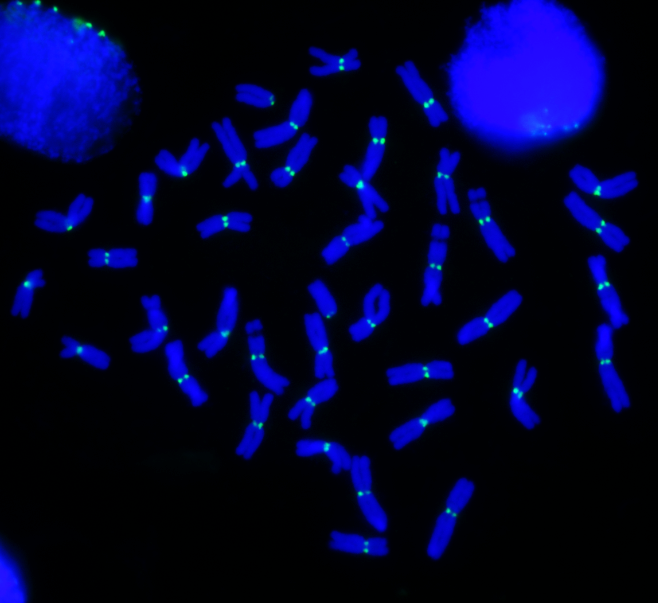** |
| --- | --- |

Figure S6. Representative chromosome spreads from 7AS (left) and 7AL (right) telosomic lines treated to detect the location of the protein CENH3 using an antibody as described in (27).

Figure S7. – IWGSC RefSeq v1.0 chromosome 7A from 338Mb-388Mb. Alignments of a single Illumina library used in the IWGSC RefSeq v1.0 assembly (1) are shown: counts of pairs where both reads align perfectly are shown in blue; reads where only a single read in the pair aligned shown in green; the sum of these two counts shown in black. Counts are in 100Kb bins. The slight drop in coverage of perfectly aligning pairs (blue bars), in tandem with a rise in only single-end alignments (green bars), occurs in the core CRW repeat region (see Fig. 6) and can be interpreted as a reduction in the short- to mid-range accuracy of the sequence in this highly repeat-dense region. Importantly this whole-genome data shows the 2Mb region deleted in the 7AL telosome (shown in Fig. 6) to be present in the whole-genome raw data.
