# Supplementary material for: Optical and physical mapping with local finishing enables megabase-scale resolution of agronomically important regions in the wheat genome
Source: Genome Biol. 2018 Aug 17;19:112. doi: 10.1186/s13059-018-1475-4 (PMC6097218; doi:10.1186/s13059-018-1475-4)
Supplement: Supplementary file 3 — a Combining the MAGIC 8-way cross 7A and Chinese Spring x Renan 7A maps [42, 43]. b Curated genetic map of 7A for anchoring the genome sequence. (ZIP 112 kb) [file 13059_2018_1475_MOESM3_ESM.zip › Additional file 3a.docx]

## **Additional file 3: Combining the MAGIC 8-way cross 7A and Chinese Spring x Renan 7A maps**

Meiotic crossing-over based genetic maps from two sources. A single reference genetic map developed from a F2 population derived from the cross between Chinese Spring (CS) and Renan (Re) and combined with a neighbor map combining the position of loci from 13 different genetic maps. The following was carried out on the stage 1 assembly. In total, 636 SSR, RFLP, STS, DArT and ISBP markers were mapped to these genetic maps and led to the anchoring of 213 contigs (225 Mb) for chromosome 7A to provide the reference CS x Renan genetic map.

A high-resolution, high-density genetic map for chromosome 7A was derived from the 8-way MAGIC population described in (42, 43) with multiple parents AC Barrie, Alsen, Baxter, Pastor, Volcani, Westonia, Xiaoyan54, and Yitpi. In total, 4173 markers were assigned to 7A based on their genotypes across three different platforms: 9K (166), 90K (1067) and GBS (2940). The number of lines genotyped ranged from 980 to 2840 depending on the platform: for the 9K SNPs, 1503 lines were genotyped; for the 90K SNPs, 2840 lines; and for the GBS markers 980. The map was constructed following a similar process to that outlined in (36) for 9K markers on the same population, utilizing the R package mpMap (42, 43) to first estimate recombination fractions between all pairs of markers, then ordering the markers based on these recombination fractions, and estimating map distances. No adjustment for segregation distortion was carried out on chromosome 7A, although markers showing excessive segregation distortion (p<1e-5 for a chi-squared test of Mendelian segregation) were removed (Additional file 8, Fig. S1). More than half of the 400 markers removed located to the centromere region.

Markers in MAGIC maps were compared with the physical contigs which formed the basic units for compiling the 7A pseudomolecule by aligning the sequences of the markers to the contigs. In total 3294 markers could be aligned uniquely to 537 contigs. The number of markers mapped to each contig ranged from 1 to 62, with the mode being 1 marker and the median being 6. For each contig containing > 1 marker, we investigated the maximum recombination fraction between any markers in the contig. Contigs which contained markers at a distance > 5 cM were examined further and grouped into two categories: containing either a single marker where an unusually inflated recombination fraction may have been due to genotyping errors or containing one or more markers which appeared to be unlinked to the other markers in the contig. In the former case, the marker was discarded from the map; in the latter, the physical contig was re-examined to determine whether the assembly potentially could be split into distinct contigs corresponding to the two groups of unlinked markers. If the physical contig evidence was ambiguous, these contigs were flagged for further investigation.

After all conflicts within contigs had been resolved, a representative marker assigned to each physical contig was selected, and these individual contig-markers were then used to align the MAGIC-7A map to the reference CS x Renan genetic map (Additional file, Fig. S2). Specifically, all contig-markers which coincided with those in the CS x Renan map were first taken as a framework map. Additional contig-markers were then added by building clusters based on recombination fractions around the framework contig-markers, and then ordering contig-markers within the clusters. Map positions were estimated based on this full set of contig-markers, which were then related back to the original full set of markers by inserting all markers within a contig at the same position as the contig-marker in the map.

For comparison purposes, a map was also created based on the contig-markers without the constraint that the order had to coincide with the CS x Renan map. For 13 markers, this resulted in noticeable differences in map positions (ranging from 10-116 cM). Upon re-examination of the physical contigs for these markers, the locations were accepted as indicating genuine differences between Chinese Spring DNA (source of the physical map and genome sequence) and the genome constitutions of the varieties used to develop the MAGIC map.

The deletion bin-based molecular marker map (https://wheat.pw.usda.gov/cgi-bin/GG3/browse.cgi?class=marker) provided 293 molecular markers and confirmed the overall structure of the chromosome 7A map. Alignments of the pseudo-molecule to the classical microsatellite-based maps were also consistent at this broad level of genome structure.

Cross-over analysis on the genotypes from the MAGIC population was based on alleles called against the IWGSC RefSeq v1.0 chromosome 7A pseudomolecule with parental origin of chromosome blocks using RABBIT (<https://github.com/chaozhi/RABBIT>). Outputs were processed with in-house scripts. Considering only parental calls with RABBIT confidence > 0.8, cross-overs were defined as the mid-point between consecutive markers (after filtering) where the parental origin was different. Only cross-overs between markers less than 5Mb apart, and for the 465 lines with the highest number of marker positions called with > 0.8 confidence, were counted.
