# Supplementary material for: Optical and physical mapping with local finishing enables megabase-scale resolution of agronomically important regions in the wheat genome
Source: Genome Biol. 2018 Aug 17;19:112. doi: 10.1186/s13059-018-1475-4 (PMC6097218; doi:10.1186/s13059-018-1475-4)
Supplement: Supplementary file 4 — DNA sequence assembly details. (DOCX 15 kb) [file 13059_2018_1475_MOESM4_ESM.docx]

## **Additional file 4: DNA sequence assembly**

For each BAC pool, filtered reads were assembled with ABySS software in multiple iterations with k-mer size ranging from 51 to 131. The “best” assembly was selected using a metric that balanced N50 scaffold size with total assembled length and number of scaffolds. This assembly formed the basis for refining the IWGSC RefSeq ver1-WGA assembly for 7A (see (1)) and establishing a WGA-independent assembly using GYDLE software (see below) to provide the basis of an independent assessment of the WGA assembly.

## Assembly finishing with GYDLE software

The set of scaffolds produced in stage1 were used as the starting point for integrating the various data sets using GYDLE software (Philippe Rigault, GYDLE Inc., Canada, [https://www.GYDLE.com/bioinformatics](https://www.gydle.com/bioinformatics); (38) (41)). An initial multiple alignment was produced using the NUCLEAR software (GYDLE Inc) as part of the hybrid assembly of the available datasets. Reprocessing of BAC pools assemblies identified BAC ends and removed low quality reads, and thus allowed BAC clones to be identified that were not true components of the respective pools. VISION software (GYDLE Inc) was used to visualize assemblies in a semi-manual curation process with assembly metrics calculated using Perl, R and Shell scripts. An iterative process provided the basis for integrating extensive mate-pair data, Bionano data and Bayer-IWGSC Whole Genome Profiling (WGP^TM^) tags *(1)*

## Annotation

Early versions of the BAC-based assemblies were annotated with TriAnnot (2), GeneID (http://genome.crg.es/software/geneid/) and MIPS pipelines (2). Final scaffolds were annotated within the IWGSC RefSeq framework (1). Regions of the chromosome of specific biological interest were selected and the automated gene models were manually examined in the web-based Apollo annotation tool (Lee et al 2012; see also (1)). In addition, all available Chinese Spring RNA-seq datasets were downloaded from EBI ArrayExpress and aligned to the chromosome (1).
