# Supplementary material for: Optical and physical mapping with local finishing enables megabase-scale resolution of agronomically important regions in the wheat genome
Source: Genome Biol. 2018 Aug 17;19:112. doi: 10.1186/s13059-018-1475-4 (PMC6097218; doi:10.1186/s13059-018-1475-4)
Supplement: Supplementary file 8 — Genome association analyses for variation in grain fructan and yield (grain number) [44–47]. (DOCX 17 kb) [file 13059_2018_1475_MOESM8_ESM.docx]

## **Additional file 8: Association analyses for grain fructan and components of yield**

Fructan content in grain

A 3 Mb region for genome sequence finishing overlapped the QTL initially defined by Huynh et al (16) for fructan content in the grain and was confirmed in the present study by reanalyzing the data using markers from a 9K SNPchip. Chemical measurements on fructan levels in the grain from 92 segregating progeny (RILs) from a Berkut x Krichauff cross were kindly provided by Prof D Mather (University of Adelaide) and combined with DNA marker assignments on 170 lines using the 9K SNPchip for QTL mapping using standard ICIM software. The replicated trait data used in the analysis is described in (16).

A large-scale association analysis was also carried out using the wheat IWGSC RefSeq v1.0 wheat sequence combined with a 90K SNP analysis of 1678 wheat lines grown under irrigated field trial and a new approach for phenotyping grain flour samples. The use of NMR spectroscopy to screen flour samples provided proton-shift spectra that show both quantitative and qualitative changes related to chemical composition (Additional file 8, Figure S4A; Simone Rochfort and Raj Pasam, in preparation). Calibration of changes in NMR spectra for variation in fructan content indicated that the 3.8 ppm proton shift classically associated with fructans (Additional file 8, Figure S4B) provided a point of reference in the association study.

Genome wide association analysis was performed using 90K SNP array with grain fructan content estimated from NMR spectra (3.8 ppm). Mixed linear model accounting for population structure with principal components and kinship matrix was used to detect significantly associated SNPs (Additional file, Figure S4B). BayesR approach, a Bayesian multilocus analysis to dissect complex traits as successfully reported in wheat (44) was also performed to pick the top significant SNPs. Both the analysis methods showed consistent results and the SNPs that are significant in both the methods were further investigated.

The 3.8 ppm window of the NMR spectra showed significant associations with SNPs in 4AL, 7AS and 7DS (Additional file 8, Figure S4B), and for 7A these associations fell within QTL interval defined in the Berkut x Krichauff bi-parental cross. An analysis of 900 wheat lines characterized using exome capture indicated that over half of the SNP variation in the QTL region associated with variation in grain fructan-levels located to one of the GH32 family genes, 6-SFT gene which in turn associated with variation in grain fructan-levels using NMR spectra for large-scale phenotyping of flour samples (3.8 ppm proton shift section of the spectra). For the homoeologous GH32 array on chromosome 7D, the most highly significant association across the entire genome was a SNP in the 1-FFT gene model. Significant associations also occurred on chromosomes 1A and 3A and were not investigated.

Yield components in RAC875/Kukri biparental map. A new genetic map of RAC875/Kukri was constructed using ASMap (41) and other functions in the R environment including R/qtl. Three sets of markers were combined and comprised: 238 SSR, 251 DArT, 28,163 SNP markers scored on 180-322 lines held at Australian Grain Technologies (AGT). The combined set of markers was curated to prepare a high resolution genetic map for QTL analysis. Lines were checked for missing data and those showing similarity for a large proportion of the genome (over 80%) were considered as clones or partial clones and removed from the map using the fixClones function in R. ASMap was used to combine the markers and reorder the map. The final map included lines that were present in all three marker sets and had less than 5% missing markers. Poor quality markers and markers containing at least two double crossovers were removed. The final map ordering was checked using ASMap and constructed with 331 DH lines and 4312 unique, high quality markers. The map included 26 linkage groups assigned to 21 wheat chromosomes. The total length of the genetic map is 2864 cM, containing 2356 unique loci with an average distance of 1.23 cM (min = 0.1 and max = 48.1 cM) between two markers.

QTL analysis of the RAC875/Kukri DH population was performed on phenotypic data collected from 24 field trials in Australia and Mexico between 2006 and 2013 (46, 47) and analysed with the WGAIM package (Taylor and Verbyla, 2011) in R (R Core Team, 2015). Traits were checked for outliers, and spatial trends were identified using the ASReml package in R, and included in the QTL model. Each trait by site combination was analysed for QTL separately, using the WGAIM options of QTL fitted as random effects at markers, with an exclusion window of 20 cM. A summary of QTL outputs is provided in Additional file 9.
