# Supplementary material for: Optical and physical mapping with local finishing enables megabase-scale resolution of agronomically important regions in the wheat genome
Source: Genome Biol. 2018 Aug 17;19:112. doi: 10.1186/s13059-018-1475-4 (PMC6097218; doi:10.1186/s13059-018-1475-4)
Supplement: Supplementary file 12 — Rice chromosome 8 centromere synteny analysis with chromosome 7A. (DOCX 14 kb) [file 13059_2018_1475_MOESM12_ESM.docx]

**Additional file 12: Rice chromosome 8 centromere synteny analysis with chromosome 7A**

Alignment and anchoring of the broad centromere region defined by the CRW sequences to the rice chromosome 8 functional centromere region (Fig. 5B) identified six highly conserved genes (TraesCS7A01G284500, TraesCS7A01G288700, TraesCS7A01G294900, TraesCS7A01G295300, TraesCS7A01G295400, TraesCS7A01G292400) that spanned both the 7A and rice8 centromere regions and were homologs to the rice genes identified by Yan et al (2008) as highly conserved across crop plants. The genomic sequence defined by both TraesCS7A01G288700 and TraesCS7A01G292400 were gapless in the IWGSC RefSeq v1.0 assembly and agreed perfectly with the equivalent region in the GYDLE and gave the same 3-D PDB fold prediction in Phyre2 (LA Kelly, http://www.sbg.bio.ic.ac.uk/phyre2/html/page.cgi) as the respective rice gene models. The genomic sequence defined by TraesCS7A01G295400 was in the GYDLE assembly, but manual inspection of the gene structure indicated the IWGSC RefSeq v1.0 annotation did not match the RNA-Seq evidence well, possibly due to an assembly difference in the TGAC assembly leading to an incorrect gene being used as reference in the IWGSC RefSeq annotation. Correcting the structure produced a gene closely matching a pentatricopeptide repeat-containing protein in rice (Phyre2 predicted the same PDB fold c4m57A for both the rice and wheat models). At the 7AS boundary of the centromere, TraesCS7A01G284500 contained 6 gaps in its introns in the IWGSC RefSeq assembly which were closed in the GYDLE assembly. This gene encoded a protein (transport protein tip20, PDB fold c3fhnA) with a high confidence hit in Phyre2. The homologous rice gene model (Os8T0318500) also encoded a protein with a high confidence match to a protein in Phyre2 (transport protein cog4, PDB fold c3hr0A) although the PDB fold was not the same. There were two gaps in a large intron of TraesCS7A01G294900 in IWGSC RefSeq v1.0, likely due to GC-content of the sequence and although the CDS had good homology to OS08G309300, no functional model was identified in Phyre2. Manual inspection of TraesCS7A01G294900 showed that it had a small final exon missing in the IWGSC Refseq annotation. At the 7AL boundary of the centromere, TraesCS7A01G295300 was only partially covered by the GYDLE assembly. The IWGSC RefSeq model had four gaps in introns which could be closed using the raw IWGSC data, though the coding part of the gene was unchanged and had a high confidence match to a membrane protein (PDB fold c3sl7B in Phyre2) for both the wheat and rice (Os08T0313200) gene models. Bionano optical maps were consistent with the genome assembly surrounding the gene.
