# Supplementary material for: Optical and physical mapping with local finishing enables megabase-scale resolution of agronomically important regions in the wheat genome
Source: Genome Biol. 2018 Aug 17;19:112. doi: 10.1186/s13059-018-1475-4 (PMC6097218; doi:10.1186/s13059-018-1475-4)
Supplement: Supplementary file 13 — Quantification of centromere fluorescence for CEN H3 antibody in situ locations. (DOCX 14 kb) [file 13059_2018_1475_MOESM13_ESM.docx]

**Additional file 13: Quantification of centromere fluorescence**

Chromosome images (Additional file 8, Figure S6) were provided in Tagged Image File (TIF) format with dimensions measuring 1392×1040 px at 150 dpi resolution. The fluorescence of the centromeres was determined by measuring their maximum pixel intensity in ImageJ v. 1.51s.

To determine a region-of-interest in each image, TIF files were opened in ImageJ and converted to an RGB stack, then a “stack to images” function was used to separate the red, green and blue channels as separate images. The blue and red channel images were discarded. The threshold of the green channel image was adjusted to values between 46 and 243 using Huang’s fuzzy thresholding method (as in ImageJ v. 1.51s). Particles were identified for analysis where their size ranged between 0 and 2000 and circularity was between 0.01 and 1.00. Particles were added to ImageJ’s “ROI Manager” before the green channel image was discarded. The original RGB image was then opened and the identified regions-of-interest used to select areas (centromeres) for analysis. Minimum and maximum values of pixel intensity were measured for the regions-of-interest. Data was saved in a comma-separated values (CSV) file and an image map was generated indicating the position of each region-of-interest for each analysis. This procedure was automated in ImageJ using a custom ImageJ Macro (IJM) script.
